# Molecular Pathogenesis of MALT Lymphoma

**DOI:** 10.1155/2015/102656

**Published:** 2015-04-01

**Authors:** Katharina Troppan, Kerstin Wenzl, Peter Neumeister, Alexander Deutsch

**Affiliations:** Division of Hematology, Department of Internal Medicine, Medical University of Graz (MUG), 8036 Graz, Austria

## Abstract

Approximately 8% of all non-Hodgkin lymphomas are extranodal marginal zone B cell lymphoma of mucosa associated lymphoid tissue (MALT), also known as MALT lymphoma, which was first described in 1983 by Isaacson and Wright. MALT lymphomas arise at a wide range of different extranodal sites, with the highest frequency in the stomach, followed by lung, ocular adnexa, and thyroid, and with a low percentage in the small intestine. Interestingly, at least 3 different, apparently site-specific, chromosomal translocations and missense and frameshift mutations, all pathway-related genes affecting the NF-*κ*B signal, have been implicated in the development and progression of MALT lymphoma. However, these genetic abnormalities alone are not sufficient for malignant transformation. There is now increasing evidence suggesting that the oncogenic product of translocation cooperates with immunological stimulation in oncogenesis, that is, the association with chronic bacterial infection or autoaggressive process. This review mainly discusses MALT lymphomas in terms of their genetic aberration and association with chronic infections and summarizes recent advances in their molecular pathogenesis.

## 1. Introduction

Approximately 8% of all non-Hodgkin lymphomas are extranodal marginal zone B cell lymphoma of mucosa associated lymphoid tissue (MALT), also known as MALT lymphoma, which was first described in 1983 by Isaacson and Wright [1, 2]. They discovered that primary low-grade gastric B cell lymphomas and immunoproliferative small intestinal disease had more histological features in common with those of mucosa associated lymphoid tissue than with peripheral lymph nodes [1]. Extranodal low-grade lymphomas arising at other mucosal organs, including the salivary gland, lung, and thyroid, showed similar histological and clinical features [3–6] establishing the term “MALT lymphoma.” MALT lymphomas arise at a wide range of different extranodal sides, including the stomach (70%), lung (14%), ocular adnexa (12%), thyroid (4%), and small intestine (including immunoproliferative small intestinal disease; 1%) [7].

The histological feature of MALT lymphoma comprises infiltration of the marginal zone and spreading diffusely into the surrounding tissue. MALT lymphoma cells share the same cytological and immunophenotypical (CD20+, CD21+, CD35+, IgM+, and IgD−) features as marginal zone B cells prompting the World Health Organization to designate this lymphoma as “extranodal marginal zone B cell lymphoma of mucosa associated lymphoid tissue (MALT lymphoma)” [8]. The lymphoma cells often resemble follicle-centre centrocytes, small lymphocytes, or the so-called monocytoid B cells. Another important histological feature of it is the presence of lymphoepithelial lesions formed by the lymphoma cell invasion of individual mucosal glands or other epithelial structures. Transformed blasts and plasma cells are scattered, present beneath the surface epithelium, possibly indicating that the MALT lymphoma might participate in the immune response. The lymphoma cells also enter the germinal centers of nonneoplastic B cell follicles—a process known as follicular colonization [9].

In the case of gastric MALT lymphoma, the disease is remarkably indolent and tends to remain localized in the stomach for long periods. The ten-year survival rate for gastric MALT lymphoma is close to 90% with a disease-free survival of approximately 70% [10, 11]. However, in rare instances, MALT lymphoma can progress and transform into aggressive high-grade tumours—extranodal diffuse large B cell lymphoma (eDLBCL)—whereby the ten-year survival rate drops to approximately 42% [10]. eDLBCLs show a more frequent BCL6 expression and have a better overall survival rate than nodal cases of DLBCL [12]. The foci of eDLBCL may be seen in MALT lymphoma, suggesting a transformation from one into the other. This has been confirmed by the demonstration of identically rearranged immunoglobulin (Ig) genes between the low- and high-grade components of the same cases [13]. In some cases of eDLBCL in which the low-grade MALT lymphoma component cannot be detected transformed MALT lymphoma is supposed to be completely overgrown by the eDLBCLs. Others are primary eDLBCLs with a germinal centered-like phenotype (CD10− and BCL6+) [10]. Transformed MALT lymphomas are CD10− and BCL2− [14], but, in contrast to MALT lymphoma, they usually express BCL6. However, there is no difference in clinical behavior between transformed MALT lymphoma and eDLBCL [10].

## 2. Genetic Aberrations

### 2.1. Translocations

**Figure 1:**
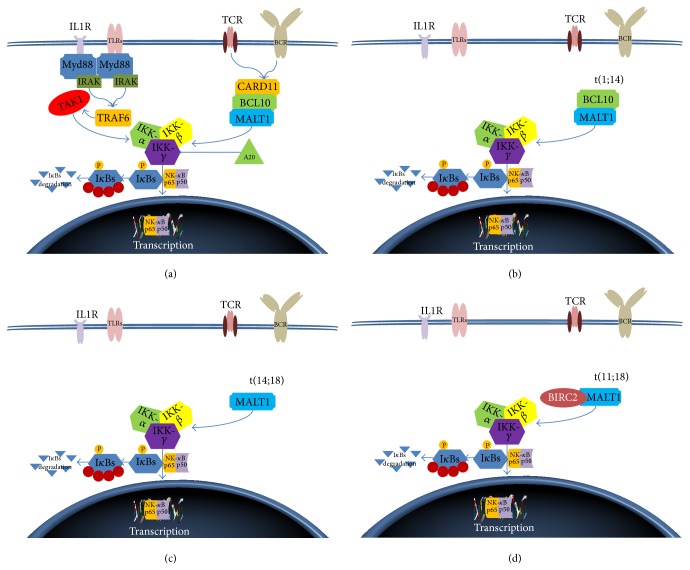
Translocations affecting the NF-*κ*B activation pathway. (a) Signaling from the TLR, IL-1R, and antigen receptor activates the canonical NF-*κ*B pathway, which is characterized by activation of the IKK complex, phosphorylation, and degradation of I*κ*B. TNFAIP3 is a negative regulator. (b) t(1;14)(p22;q32) results in the nuclear overexpression of the BCL10 protein. It is believed to form oligomers through its CARD domain and so it triggers MALT1 oligomerization and aberrant NF-*κ*B activation. (c) t(14;18)(q32;q21) causes overexpression of MALT1. It is thought that it oligomerizes through interaction with BCL10 causing NF-*κ*B activation. (d) t(11;18)(q21;q21), the BIR domain of the BIRC2-MALT1, mediates self-oligomerization leading to an activation of NF-*κ*B. TLR: Toll-like receptor; IL-1R: interleukin-1 receptor; BCR: B cell receptor; TCR: T cell receptor; RIP1: receptor interacting protein 1; TRAF: TNF-associated factor; TAK1: transforming growth factor beta activated kinase 1; TAB: TAK binding protein; IKK: inhibitor of NF-*κ*B kinase; I*κ*B: inhibitor of NF-*κ*B.

There are four main recurrent chromosomal translocations associated with the pathogenesis of MALT lymphomas: t(1;14)(p22;q32), t(11;18)(q21;q21), t(14;18)(q32;q21), and t(3;14)(p14.1;q32) [15–18]. The frequency of genetic aberrations is also dependent on the primary site of disease. Translocation t(11;18)(q21;q21) was mainly found in pulmonary and gastric tumors, whereas t(14;18)(q32;q21) was most detected in ocular adnexal, orbit, skin, and salivary gland MALT lymphoma [19] (Figure 1).

The t(1;14)(p22;q32) translocation occurs in 1% to 2% of MALT lymphomas and has been reported in the stomach, lung, and skin [19]. The entire coding sequence of the* BCL10* gene on chromosome 1 is relocated to the immunoglobulin heavy chain (IgH) enhancer region on chromosome 14 resulting in the nuclear overexpression of the BCL10 protein. The t(1;14)(p22;q32) translocation has exclusively been reported in MALT lymphoma, and these cases typically display additional genomic alterations. Patients with advanced stage MALT lymphoma exhibit this translocation and do not respond to* Helicobacter pylori *(*H. pylori*) eradication [20].

The t(14;18)(q32;q21) translocation occurring in 15% to 20% of MALT lymphomas brings the* MALT1* gene under the transcriptional control of the IgH enhancer region on chromosome 14 [17]. This translocation occurs more frequently in nongastrointestinal MALT lymphomas. In contrast to t(11;18)(q21;q21), the t(14;18)(q32;q21) is frequently associated with other cytogenetic abnormalities [19]. t(14;18)(q32;q21) positive cases also show an overexpression of the BCL10 protein but display cytoplasmatic localization in contrast to t(1;14)(p22;q32) and t(11;18)(q21;q21) positive MALT lymphomas [21, 22].

The t(11;18)(q21;q21) translocation is the most common translocation, occurring in 15–40% of all MALT lymphomas [16, 19]. This translocation is restricted to MALT lymphomas and has not been found in nodal or splenic marginal zone lymphomas (MZL). In most of these translocation-positive cases, it is the sole chromosomal aberration and only in exceptional cases has it been detected in de novo DLBCL arising at mucosal sites [23–25]. The t(11;18)(q21;q21) has been found in MALT lymphomas at a number of different anatomic sites, including lung, stomach, intestine, and, less commonly, skin, orbit, and salivary gland [19, 26]. It has also been associated with cases that do not respond to* H. pylori* eradication [27, 28] and is rarely seen in transformed MALT lymphomas [25]. The t(11;18)(q21;q21) translocation represents the fusion of the apoptosis inhibitor 2—named* BIRC2* (API2)—gene on chromosome 11 and the MALT lymphoma associated translocation 1 (*MALT1*) gene on chromosome 18 [29]. Breakpoints observed in this translocation are clustered in the region of intron 7 and exon 8 of the* BIRC2* gene and introns 4, 6, 7, and 8 of the* MALT1* gene. High frequencies of deletions and duplications in both genes are also found, which implies that multiple double-strand DNA breaks (DSBs) must have occurred during the translocation process appearing as a result from illegitimate nonhomologous end joining after DSBs [30]. The resulting fusion transcript always comprises the N-terminal* BIRC2* with three intact baculovirus inhibitor of apoptosis repeat (BIR) domains and the C-terminal* MALT1* region containing an intact caspase-like domain [16, 26, 31]. t(11;18)(q21;q21) cases show a nuclear overexpression of the BCL10 protein [20].

The t(3;14)(p14.1;q32) translocation has been most recently described and establishes the juxtaposition of the transcription factor FOXP1 next to the enhancer region of the IgH chain genes [18]. Overexpression of FOX1P analysed by chromatin immunoprecipitation in lymphoma cells demonstrates that FOX1P acts as transcriptional repressor of multiple proapoptotic genes repressing caspase-dependent apoptosis [32].

The occurrence of the recurrent translocations t(1;14)(p22;q32), t(14;18)(q32;q21), and t(11;18)(q21;q21) in MALT lymphoma, constitutively activating the NF-*κ*B pathway by the association of* BCL10* and* MALT1* in malignant lymphocytes, defines this pathway as an oncogenic event [33, 34]. Physiologically, BCL10 binds to the Ig-like domain of MALT1, and this binding induces the MALT1 oligomerization [33]. The BCL10-MALT1 complex promotes the ubiquitylation of I*κ*B kinase-*γ* and NF-*κ*B is released to translocate into the nucleus and to transactivate genes, such as those encoding factors for cytokines and growth factors for cellular activation, proliferation, and survival [35]. In MALT lymphoma with t(1;14)(p22;q32), BCL10 is believed to form oligomers through its CARD domain without the need for upstream signaling and thus triggers the MALT1 oligomerization and aberrant* NF-κB* activation. In lymphoma cases with t(14;18)(q32;q21),* MALT1* is overexpressed. MALT1 does not possess a structural domain mediating self-oligomerization and it does not activate NF-*κ*B* in vitro* [33, 34]. It seems likely that MALT1 interacts with and stabilizes BCL10, causing its accumulation in the cytoplasm of t(14;18)(q32;q21) positive tumor cells resulting in oligomerization of MALT1 and activation of NF-*κ*B [36]. In t(11;18)(q21;q21) positive MALT lymphomas the BIR domain of the BIRC2-MALT1 mediates self-oligomerization, which in turn leads to NF-*κ*B activation [37, 38].

However, two different transgenic mice—overexpressing either of the two translocations, BCL10 or BIRC2-MALT1, seen frequently in MALT lymphomas—develop splenic marginal zone hyperplasia, but not lymphoma [39, 40]. However, Sagaert et al. [41] reported lymphoma development when BIRC2-MALT1 mice were exposed to antigen stimulation. Altogether, these data indicate that in MALT lymphoma chromosome translocations alone are not sufficient for full malignant transformation. Cooperation with a chronic infectious process seems to be necessary for lymphomagenesis. Recently, a novel molecular mechanism of the BIRC2-MALT1 fusion protein has been identified [42]. Nie et al. demonstrated that the tumor suppressor gene* LIMA1* binds* BIRC2* and is proteolytically cleaved by* MALT1* through its paracaspase activity. This cleavage generates a LIM domain—only (LMO)—containing fragment with oncogenic properties* in vitro* and* in vivo*.

### 2.2. Numeric Chromosomal Aberration: Trisomies and Deletions

Other cytogenetic alterations include trisomies 3, 12, and/or 18, which are present as a sole abnormality in 22% of the cases, but they are often associated with one of the four main translocations described above [19].

Taji et al. detected trisomy 3 as the most common aberration in gastrointestinal MALT lymphomas with a frequency of up to 35% [43]. Partial trisomies of chromosomes 3 and 18 also have been observed, as published by Krugmann et al. [44]. In contrast, Ott et al. reported an incidence of only 20% trisomy 3 in low-grade MALT lymphoma and an even lower rate in high-grade ones [26]. The genetic mechanism by which trisomy 3 may contribute to lymphomagenesis has not yet been experimentally addressed. However, an increased gene dosage effect resulting from higher copy numbers of genes relevant to lymphoma development is likely to explain the biological consequences underlying chromosomal trisomies. Several promising candidate genes are located on chromosome 3 and have been implicated in lymphomagenesis, such as the protooncogene* BCL6* and the transcription factor FOXP1 [24]. One of our previous studies describes CCR4—a chemokine receptor genomically located on chromosome 3 (3p24)—highly expressed in trisomy 3 + MALT lymphoma whereas transcripts for this chemokine receptor were missing in trisomy 3− MALT lymphomas [45].

Apart from the typical chromosomal translocations,* TNFAIP3* (*A20*) has been identified as frequently deleted in ocular adnexal MALT lymphoma as detected by array comparative genomic hybridization [46–48]. As an important player in the NF-*κ*B pathway by various mechanisms,* TNFAIP3* acts as a tumor suppressor gene in various lymphoma subtypes. In ocular adnexal MALT lymphoma, complete* TNFAIP3* inactivation is associated with poor lymphoma-free survival [46, 49].* TNFAIP3* deletion occurred in MALT lymphoma of the ocular adnexa (19%), salivary gland (8%), thyroid (11%), and liver (0.5%), but not, or at almost undetectable frequencies, in the lung, stomach, and skin [46, 50]. However,* TNFAIP3* inactivation alone is not sufficient for malignant transformation but nevertheless may represent a promising future therapeutic target [51].

### 2.3. Somatic Mutations

To our knowledge, the number of studies investigating somatic mutations in MALT lymphoma is low and a whole genome sequencing approach has not yet been done. Our group reported somatic missense mutations in* PIM1* and* cMyc* in 46% and 30% of MALT lymphomas (gastric and extragastric origin) and in 30% and 41% of transformed MALT lymphomas and 72% of primary cutaneous marginal zone B cell lymphomas (PCMZL) [52], considered as integral part of MALT lymphomas [53, 54]. Du et al. [55] detected missense and frameshift mutations in p53 in 20.8% of MALT lymphoma and 30% of transformed MALT lymphoma (both mainly of gastric origin). Mutation analysis of NF-*κ*B signal pathway-related genes—*TNFAIP3*,* Card11*,* CD79B*, and* Myd88*, known to be frequently mutated in aggressive lymphomas [56–59]—demonstrated missense or frameshift mutations in 6% of MALT lymphoma cases in the* Myd88* locus and in 28.6% of ocular adnexal MALT lymphomas mutations in the* TNFAIP3* locus [49, 60, 61].

Liu et al. [62] reported that* Card11* and* CD79B* were not affected in their cohort of ocular adnexal MALT lymphomas.

These genetic lesions are not restricted to MALT lymphoma. Rinaldi et al. performed a comprehensive analysis of genomic DNA copy number changes in more than 200 samples of MZL and demonstrated a distinct distribution of lesions in different subtypes (MALT lymphoma, nodal MZL, and splenic MZL). Whereas 3q and 18q gains were common in all three subtypes, del(6q23)(*TNFAIP3*) could be used for differentiation between MALT lymphoma and splenic MZL [63].

To investigate the role of TNFAIP3 as tumor suppressor in MZL, Novak et al. analyzed 32 MZL including 11 extragastric MALT lymphomas by SNP-array [64]. They were able to identify somatic mutations in four of 11 extragastric MALT lymphomas, as well as a genetic loss of TNFAIP3 in two of the four somatically mutated MALT lymphomas. Interestingly, no* PRDM1* (*Blimp1*) deletions were detected in samples with* TNFAIP3* deletion (Table 1).

## 3. The Connection to Long-Lasting Chronic Infection

Gastric MALT lymphoma is strongly associated with the chronic infection* H. pylori*, which is an association that satisfies Koch's postulates for an etiologic agent [65]. Other infectious associations, though not entirely fulfilling these criteria, have been reported for* Borrelia burgdorferi* (skin) [66],* Campylobacter jejuni* (intestine) [67], and the hepatitis C virus (splenic marginal zone lymphoma) [68]. Other chronic inflammatory reactions or autoimmune diseases have been further associated with MALT lymphoma, including Sjogren's disease [69]. In ocular adnexal MALT lymphoma especially, representing 5–15% of all extranodal lymphomas, the occurrence of* Chlamydia psittaci* is of special interest. Ferreri et al. [70] demonstrated an association between ocular adnexal MALT lymphoma and infection with* Chlamydia psittaci* in an Italian patient cohort. The presence of* Chlamydia psittaci* DNA was detected in 80% of lymphoma samples. Moreover, bacterial DNA was found in 43% of peripheral blood mononuclear cells from patients, but not in healthy donors. More than 80% of these patients achieved lymphoma after* Chlamydia psittaci* was successfully eradicated by doxycycline administration [71]. In a large study of 142 cases, Chanudet et al. [72] described an overall prevalence (22%) of* Chlamydia psittaci* infection in ocular adnexal MALT lymphoma, but with marked geographic variation, the highest incidences being in Germany (47%), the East Coast of the United States (35%), and Netherlands (29%). In our Austrian study, we detected* Chlamydia psittaci* in 7 out of 13 samples of ocular adnexal MALT lymphoma, in contrast to only one of 17 gastrointestinal specimens tested positive [73]. A subsequent study by our group in 47 nongastrointestinal MALT lymphomas demonstrated 13 (28%) to be positive for* Chlamydia psittaci* DNA compared to only 4 (11%) of 37 nonmalignant control samples (*P* = 0.09).* Chlamydia psittaci* was detected at variable frequencies in MALT lymphomas of different sites with up to 100% frequency in pulmonary MALT lymphomas, suggesting a possible causal involvement of this pathogen [74] in MALT lymphomagenesis.

A role for antigen-driven clonal expansion of the lymphoma cells is shown in the evidence of an ongoing somatic hypermutation in the Ig* V* genes [75]. The involvement of antigens is further supported by evidence of clonal evolution within the tumor, suggesting selective pressure to increase affinity of the immunoglobulin for antigens [76]. The early stages of gastric lymphoma development may be facilitated by antigen-driven T cells specific for the* H. pylori* organism [77] and the eradication of the infection causing a cure rate up to 75% is consistent with this postulate [78]. However, even less is known about the role of the host immune response, as demonstrated by the fact that only a minority of infected patients develop gastric MALT lymphoma [79]. MALT lymphomagenesis may also correlate with different cytokines and HLA polymorphisms [80, 81].

## 4. Pathogenesis of MALT Lymphomas

The evolution of gastric MALT lymphoma is a multistage process starting with the infection of* H. pylori* resulting in the recruitment of B and T cells and other inflammatory cells to the gastric mucosa. The infiltrated B cells are stimulated by the* H. pylori*-specific T cells and undergo malignant transformation due to the acquisition of genetic abnormalities. One example is the association between the* H. pylori* infection and gastric MALT lymphoma, in which* H. pylori* stimulates tumor cell growth when coincubated with helper T cells [77]. Epithelial cells are activated by chronic infectious stimuli, expressing high levels of HLA-DR and costimulatory molecules, including CD80, on their surface. These cells may be able to present antigens along with HLA molecules to T cells. CD40 ligand molecules expressed on the activated T cells can react with the CD40 molecule on B cells, upregulating B cell expression of CD80. This surface protein can react with the CD28 molecule on CD4 T cells, strongly activating the latter. Activated CD4 T cells can stimulate B cells through CD40L-CD40 interaction, in conjunction with the action of various cytokines and chemokines. This interaction among epithelial cells, T cells, and B cells may allow these cells to survive cooperatively in lymphoepithelial lesions and not to undergo apoptosis [82]. Lymphoepithelial lesions (LELs) are thought to be the origin of lymphomas [83]. The transition from polyclonal to a monoclonal lesion is facilitated by chronic stimulation with exogenous or autoantigens, thereby increasing the frequency of their transformation [84–86]. MALT lymphoma with* H. pylori*-dependent alterations like trisomies 3, 12, or 18 can progress and become* H. pylori*-independent. Eventually it may transform into high-grade tumors following the mechanism described above. Complete inactivation of the tumor suppressor gene* P53*, homologous deletion of the* P16* gene, and chromosomal translocation of cMYC and BCL6 are associated with the transformation of MALT lymphoma [55, 87–90]. MALT lymphomas, devoid of t(11;18)(q21;q21) with an amplification at 3q27, are prone to high-grade transformation [91]. On the other hand, MALT lymphomas with t(11;18)(q21;q21) are* H. pylori*-independent but rarely transform to aggressive lymphoma [7].

## 5. MALT Lymphomas Are Targeted by the Aberrant Somatic Hypermutation

Aberrant somatic hypermutation (ASHM), which was first described in DLBCL, has been identified as a crucial contributor to the development of lymphoid neoplasm. In DLBCL, the physiological process of somatic hypermutation, occurring in the rearranged* V* genes to generate antibody diversity of germinal-centre B cells and of all germinal-center-derived B cell tumors [92, 93], aberrantly targets the 5′ sequences of several protooncogenes relevant to lymphomagenesis, including PIM1, PAX5, RhoH/TTF, and cMYC. This phenomenon occurs in >50% of DLBCL but is rare in indolent lymphomas [94–97]. The pathogenesis of most B cell non-Hodgkin lymphomas (B NHL) is associated with distinct genetic lesions, including chromosomal translocations and ASHM, which arise from mistakes during class switch recombination (CSR) and SHM occurring in the germinal centre [92, 93, 98, 99]. Activation-induced cytidine deaminase (AID) is an enzyme required for SHM and CSR, and mistargeting of AID to known protooncogenes linked to B cell tumorigenesis in germinal-center B cells combined with a breakdown of protective high fidelity repair mechanism has been shown to be a principal contributor to the pathogenesis of B NHL [98, 99]. Our group described that in 13 (76.5%) of 17 cases of MALT lymphomas and all 17 (100%) cases of extranodal DLBCL—still exhibiting a low-grade MALT lymphoma component (the so-called transformed MALT lymphoma)—were targeted by ASHM. Expression levels of AID were associated with the mutational load caused by ASHM [54]. Additionally, 8 of 11 PCMZL (72.7%)—considered as part of the group of MALT lymphomas [53]—displayed molecular features typical for ASHM [51]. Interestingly,* H. pylori* infection upregulates AID expression via NF-*κ*B resulting in gastric cells* in vitro* and* in vivo*. The* H. pylori*-mediated AID upregulation causes an accumulation of p53 mutation* in vitro* [100]. Thus, it might be speculated that* H. pylori* infection might cause an upregulation of AID in B cells and that mistargeting of this enzyme to protooncogenes induces genetic alterations relevant to MALT lymphomagenesis.

## 6. BCR Signaling in MALT Lymphoma

The BCR signaling pathway, physiologically involved in the development and differentiation of normal B cells, has been identified as playing a crucial role in lymphomagenesis and acting as an important target for therapeutic interventions [101]. The activation of this pathway is driven by multiple factors, including chronic exposure to antigens like* H. pylori*. Together with the chronic inflammatory status caused by* H. pylori*, antigen drive/stimulation may contribute to MALT lymphomagenesis; however, a direct connection between the BCR pathway and* H. pylori* has not been identified [102]. Nonetheless, early stage* H. pylori*-positive MALT lymphoma can be cured by eradicating the* H. pylori* infection alone, supporting a causative role [103].

The downstream target of the BCR signaling, NF-*κ*B, can be activated independent of BCR signaling by the MALT1 fusion protein and BCL10 overexpression [101]. MALT1 fusion protein is a result of t(11;18)(q21;q21), occurring in more advanced cases of MALT lymphoma [29]. Many MALT lymphomas require MALT1 for NF-*κ*B activation. The importance of MALT1 protease activity was shown recently by the dependency of NF-*κ*B-addicted B cell lymphomas on this proteolytic activity. Therapeutic targeting of MALT1 protease activity might therefore become a promising approach for the treatment of MALT lymphomas and other B cell lymphomas associated with deregulated NF-*κ*B signaling [104]. Consequently, MALT lymphoma, harboring these translocations, shows impaired response to antibiotic eradication therapy [105].

## 7. Chemokine Receptors in MALT Lymphomas

Chemokines, also known as proinflammatory chemotactic cytokines, represent a large superfamily of peptides with diverse biological functions. Chemokines interact with a target cell by binding to the chemokine receptors. There exist numerous chemokines and chemokine receptors, but no single chemokine is assigned to a single receptor. Chemokine signaling can coordinate cell movement during inflammation, as well as the homeostatic transport of hematopoietic stem cells, lymphocytes, and dendritic cells [106–108]. The homeostatic transport of precursor B cells to secondary lymphoid tissue is essential for B cell development. CCR6, CCR7, CXCR3, CXCR4, and CXCR5 play a crucial role in this homing process; therefore these five chemokine receptors are called B cell homeostatic chemokine receptors [109–111]. The group of activation dependent chemokine receptors, which are expressed on effector leukocytes (including activated effector/memory T cells), plays an essential role in inflammation processes responsible for migration towards chemokines produced by inflamed cells [106]. Our expression analysis of 19 well-characterized chemokine receptors in MALT lymphomas demonstrated a distinct signature of chemokine receptor expression in extragastric MALT lymphomas compared to gastric MALT lymphomas. In comparing gastric to extragastric MALT lymphomas, the upregulation of CXCR1 and CXCR2 accompanied by downregulation of CCR8 and CX3CR1 and loss of XCR1 expression in extragastric MALT lymphomas appear to be key determinants for the site of origin of MALT lymphomagenesis [45]. In our second study on the chemokine receptor in MALT lymphomas, the CXCR4 expression was missing in gastric MALT lymphomas or gastric extranodal DLBCL compared to nodal lymphomas, nodal MZL, and nodal DLBCL, which exhibited a strong expression [112] indicating that CXCR4 expression is associated with nodal manifestation. Additionally, we found that CXCL12 and CXCR7—a CXCRL12 receptor—were upregulated during the progression of gastric MALT lymphomas to gastric eDLBCL [112], suggesting at least in part an implication of this signaling pathway in high-grade transformation of gastric MALT lymphomas.

## 8. Conclusion

MALT lymphomas represent a heterogeneous group of lymphoid neoplasms with a large number of different genetic alterations depending on the site of origin [15–19]. Interestingly, most of the genetic alterations affect NF-*κ*B signal pathway-related genes causing constitutive activation of the NF-*κ*B pathway [33, 34, 36–38]. This observation is substantiated by the fact that treatment with bortezomib [113, 114]—a proteasome inhibitor with inhibitory effects on the NF-*κ*B signal pathway [115]—induces complete remissions in a substantial proportion of MALT lymphoma patients. To our knowledge, activated NF-*κ*B is also found in MALT lymphoma patients without any translocation or mutation in any of the NF-*κ*B signal pathway-related genes, so more studies on genetic alterations with a whole genome/transcriptome approach are needed to clarify the molecular mechanism of NF-*κ*B activation.

The development of MALT lymphoma is strongly associated with chronic infection by pathogens or autoantigens [65, 66, 70–72]. Eradication of the bacterial pathogen by antibiotics causes remission in the majority of MALT lymphoma patients [71, 78]. However, from our perspective, more refined studies on bacterial and viral pathogens using a next generation sequencing approach and additionally analyzing the potentially restricted usage of variable genes of the immunoglobulin genes will further clarify the causal relationship of MALT lymphomagenesis and chronic infectious or inflammatory processes.

## Figures and Tables

**Table 1:** Genetic alterations in MALT lymphomas.

Name	Type	Affected gene	NF-*κ*B activation	Subtype of MALT lymphoma mainly involved
t(1;14)(p22,q32)	Translocation	*BCL10 *	Yes	Stomach, lung, and skin
t(11;18)(q21,q21)	Translocation	*BIRC2*, *MALT1 *	Yes	Pulmonary, gastric
t(14;18)(q32,q21)	Translocation	*MALT1 *	Yes	Ocular adnexa, orbit, skin, and salivary glands
t(3;14)(p14.1,q32)	Translocation	*FOXP1 *	No	

Trisomy 3	Trisomy	FOXP1^*^ BCL6^*^	No No	Gastrointestinal
Trisomy 12	Trisomy	Unknown	No	
Trisomy 18	Trisomy	Unknown	No	

TNFAIP3	Deletion	*TNFAIP3 *	Yes	Ocular adnexal, salivary gland, thyroid, and liver

PIM1	Mutation	*PIM1 *	No	Gastric, extragastric
cMyc	Mutation	*cMyc *	No	Gastric, extragastric
P53	Mutation	*P53 *	No	
Myd88	Mutation	*Myd88 *	Yes	Ocular adnexal

P16	Hypermethylation	*P16 *	No	
P57	Hypermethylation	*P57 *	No	
TNFAIP3	Hypermethylation	*TNFAIP3 *	No	Ocular adnexal, salivary, and thyroidal glands

^*^The two potentially affected genes.
